# Flow organization and heat transfer in turbulent wall sheared thermal convection

**DOI:** 10.1017/jfm.2020.378

**Published:** 2020-06-17

**Authors:** Alexander Blass, Xiaojue Zhu, Roberto Verzicco, Detlef Lohse, Richard J. A. M. Stevens

**Affiliations:** 1 Physics of Fluids Group, Max Planck Center for Complex Fluid Dynamics,J. M. Burgers Center for Fluid Dynamics and MESA+ Research Institute, Department of Science and Technology, University of Twente, P.O. Box 217, 7500 AE Enschede,The Netherlands; 2 Center of Mathematical Sciences and Applications, School of Engineering and Applied Sciences, Harvard University, Cambridge, MA 02138, USA; 3 Dipartimento di Ingegneria Industriale, University of Rome ‘Tor Vergata’, Via del Politecnico 1,Roma 00133, Italy; 4 Gran Sasso Science Institute - Viale F. Crispi, 7 67100 L’Aquila, Italy; 5 Max Planck Institute for Dynamics and Self-Organization, Am Fassberg 17, 37077 Göttingen, Germany

**Keywords:** turbulent convection, heat transfer, Rayleigh–Bénard convection, Couette flow

## Abstract

We perform direct numerical simulations of wall sheared Rayleigh–Bénard convection for Rayleigh numbers up to 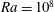, Prandtl number unity and wall shear Reynolds numbers up to 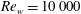. Using the Monin–Obukhov length 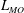 we observe the presence of three different flow states, a buoyancy dominated regime (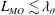; with 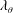 the thermal boundary layer thickness), a transitional regime (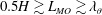; with 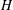 the height of the domain) and a shear dominated regime (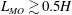). In the buoyancy dominated regime, the flow dynamics is similar to that of turbulent thermal convection. The transitional regime is characterized by rolls that are increasingly elongated with increasing shear. The flow in the shear dominated regime consists of very large-scale meandering rolls, similar to the ones found in conventional Couette flow. As a consequence of these different flow regimes, for fixed 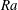 and with increasing shear, the heat transfer first decreases, due to the breakup of the thermal rolls, and then increases at the beginning of the shear dominated regime. In the shear dominated regime the Nusselt number 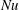 effectively scales as 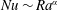 with 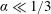, while we find 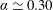 in the buoyancy dominated regime. In the transitional regime, the effective scaling exponent is 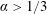, but the temperature and velocity profiles in this regime are not logarithmic yet, thus indicating transient dynamics and not the ultimate regime of thermal convection.

## Graphical abstract

**Figure:**
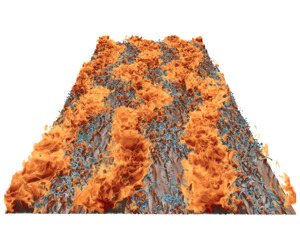

## 1 Introduction

Rayleigh–Bénard (RB) convection, i.e. the flow in a box heated from below and cooled from above, is one of the paradigmatic fluid dynamical systems (Ahlers, Grossmann & Lohse 2009; Lohse & Xia 2010; Chilla & Schumacher 2012; Xia 2013). The dynamics of RB convection driven by an imposed temperature difference is controlled by the Rayleigh number 1.1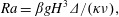 which is the non-dimensional temperature difference between the horizontal plates, and the Prandtl number 1.2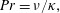 which is the ratio between kinematic viscosity and thermal diffusivity. In (1.1) and (1.2), 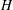 is the distance between the plates, 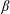 the thermal expansion coefficient of the fluid, 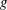 the gravitational acceleration, 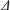 the temperature difference between the top and bottom plate and 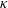 and 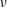 the thermal diffusivity and kinematic viscosity, respectively. Length scales are normalized by 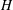 unless specified otherwise. While for purely thermally driven flows 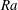 and 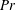 are enough to characterize the flow, when an external shear is introduced an additional control parameter is needed. In this work, we analyse the effect of the wall shear Reynolds number 1.3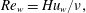 where 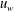 is the velocity of the wall. The ratio between buoyancy and shear driving can be expressed using the bulk Richardson number 1.4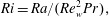 which can be seen as alternative control parameter for either 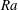 or 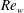.

Important responses of the system are the Nusselt number 1.5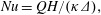 which is the dimensionless vertical heat flux, the friction Reynolds number 1.6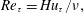 and the skin friction coefficient 1.7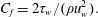 Here, 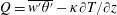 is the constant vertical heat flux, with 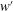 and 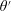 the fluctuations for wall-normal velocity and temperature, respectively, and 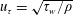 the friction velocity, with 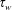 the mean wall shear stress and 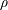 the density of the fluid.

For pure RB convection 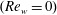 and strong enough thermal driving, i.e. high enough 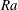, the flow in the bulk region becomes fully turbulent. For even stronger thermal driving, beyond some critical 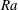 number 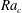, the boundary layers also become turbulent, and the system reaches the regime of so-called ultimate convection (Kraichnan 1962; Grossmann & Lohse 2000, 2001, 2011). This ultimate regime sets in when the shear Reynolds number at the boundary layers is sufficiently high so that the boundary layer becomes turbulent, leading to a strong increase in the heat transport, quantified by the Nusselt number.

Ahlers *et al.* (2012) found that the transition to the ultimate regime sets in around 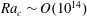. While in the classical regime one generally finds 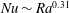, in the experimentally accessible ultimate regime an effective scaling of 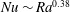 is observed, in agreement with theoretical predictions (Grossmann & Lohse 2011).

The transition to the ultimate regime has also been observed in direct numerical simulations (DNS) of two-dimensional RB convection (Zhu *et al.* 2018*a*). In Taylor–Couette flow, which is an analogous system, experiments and DNS have observed the ultimate regime as well (Grossmann, Lohse & Sun 2016). However, so far, the ultimate regime has not yet been achieved in DNS of three-dimensional RB flows (Stevens, Verzicco & Lohse 2010; Stevens, Lohse & Verzicco 2011) as the required computational time to achieve this is still out of reach. Here, in an attempt to trigger the transition to the ultimate regime, we add a Couette type shearing to the RB system to increase the shear Reynolds number in the boundary layers.

In Couette flow the top and bottom walls move in opposite directions (Thurlow & Klewicki 2000; Barkley & Tuckerman 2005; Tuckerman & Barkley 2011) with constant 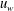 and just as in other examples of wall-bounded turbulence (Jiménez 2018; Smits, McKeon & Marusic 2011; Smits & Marusic 2013) the flow is dominated by elongated streaks, which have been observed in experiments (Kitoh & Umeki 2008) and DNS (Lee & Kim 1991; Tsukahara, Kawamura & Shingai 2006), even at relatively low shear Reynolds numbers (Chantry, Tuckerman & Barkley 2017). Pirozzoli, Bernardini & Orlandi (2011, 2014) and Orlandi, Bernardini & Pirozzoli (2015) showed that these streaks in Couette flow have much longer characteristic length scales than in Poiseuille flow, where the flow is forced by a uniform pressure gradient rather than by wall shear. Rawat *et al.* (2015) showed that these large-scale flow structures even survive when the small-scale structures are artificially suppressed. Recently, Lee & Moser (2018) found that the streak length increases with increasing shear Reynolds number and that some correlation in the streamwise direction remains visible up to a length of almost 160 times the distance between the plates.

Investigating the interaction between buoyancy and shear effects is also very important to better understand oceanic and atmospheric flows (Deardorff 1972; Moeng 1984; Khanna & Brasseur 1998). For example, early experiments on sheared thermal convection by Ingersoll (1966) and Solomon & Gollub (1990) showed the appearance of large-scale structures. Fukui & Nakajima (1985) showed that in channel flow unstable stratification increases the longitudinal velocity fluctuations close to the wall, while in the bulk region, the temperature fluctuations are drastically lowered. Furthermore, recent experiments by Shevkar *et al.* (2019) investigated the plume spacing in sheared convection and found a scaling law that connects the mean spacing of the plumes with 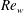, 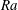 and 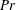.

Early simulations of sheared convection were performed by Hathaway & Somerville (1986) and Domaradzki & Metcalfe (1988) for 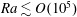. Domaradzki & Metcalfe (1988) found that in Couette–RB flow the addition of shear at low 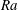 initially increases the heat transport. However, for 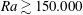 the heat transport decreases as the added shear breaks up the large-scale structures. More recently, Scagliarini, Gylfason & Toschi (2014), Scagliarini *et al.* (2015) showed that also in Poiseuille–RB the heat transfer first decreases when the applied pressure gradient is increased. The reason is that for intermediate forcing the longitudinal wind disturbs the thermal plumes, which therefore lose their coherence. Only with a strong enough pressure gradient is a heat transfer enhancement found.

The Richardson number quantifies the ratio between the buoyancy and shear forces in Couette–RB and Poiseuille–RB based on the applied temperature difference and wall shear Reynolds number. Another way to quantify the ratio between buoyancy and shear forces is to determine the Monin–Obukhov length (Monin & Obukhov 1954; Obukhov 1971) 1.8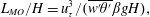 which indicates up to which distance from the wall the flow is dominated by shear. Note that 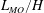 is a response parameter, in contrast to 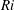, which is a control parameter. Pirozzoli *et al.* (2017) found that the Monin–Obukhov length scales as 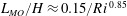 for channel flow with unstable stratification. In appendix C we show that here for Couette–RB 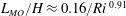.

**Figure 1.**
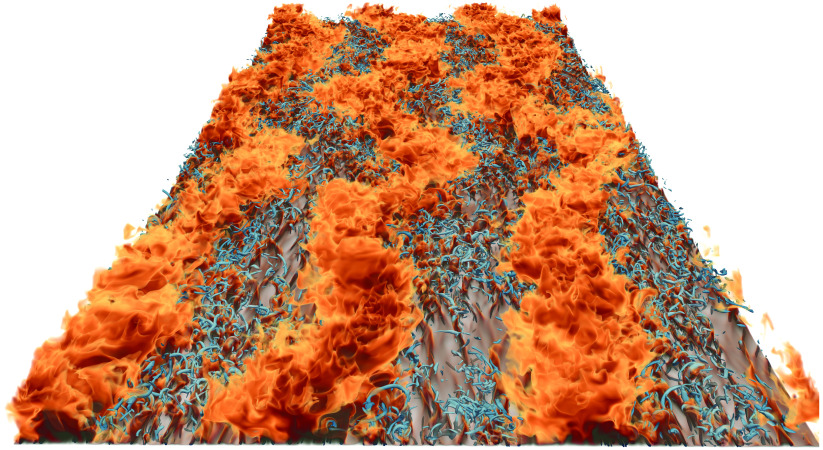
Volume rendering of the thermal structures rising from the heated plate in a simulation with 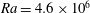 and 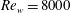. The plate dimensions are 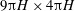, in the streamwise and spanwise directions, respectively, where 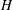 is the distance between the plates. The red colours show hot thermal structures emerging from the hot plate, while the blue structures show vorticity formations in the flow. For further details of the flow visualization, please see Favre & Blass (2019).

**Figure 2.**
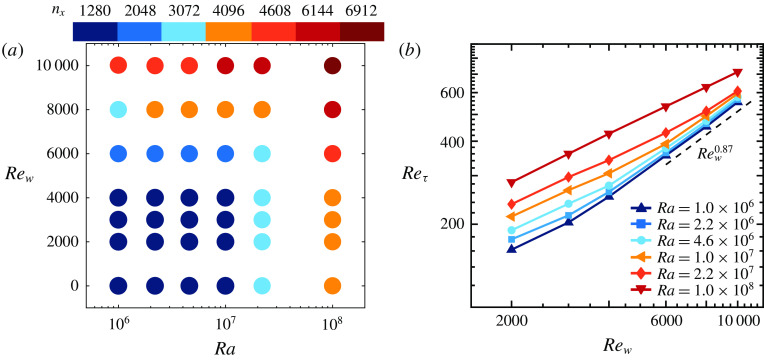
(*a*) Streamwise (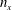) resolution used in the simulations as function of 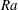 and 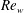, see table 2 for details. (*b*) 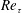 versus 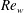 obtained from the simulations. In agreement with Pirozzoli *et al.* (2014) and Avsarkisov, Hoyas & García-Galache (2014) we find that 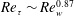 for large 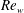.

In this study, we investigate the effect of an additional Couette type shearing on the heat transfer in RB convection in an attempt to trigger the boundary layers to become fully turbulent and hence observe the transition to the ultimate regime. Figure 1 shows a flow visualization of the temperature field obtained from one of our simulations, which reveals large-scale meandering streaks that are formed near the hot plate. We performed simulations over a wide parameter range, spanning 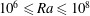 and 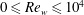, while 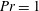 has been used in all cases, see figure 2(*a*). Despite the very strong forcing for the largest 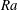 and 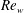, we did not achieve ultimate turbulence. We were limited by our requirement of using large domain sizes, as recommended by Pirozzoli *et al.* (2017), to ensure convergence of the main flow properties.

The remainder of this manuscript is organized as follows. In § 2 we present the simulation method. We discuss the heat transfer and skin friction measurements in §§ 3.1 and 3.2, respectively. A discussion of the identified flow regimes is given in § 4. The concluding remarks follow in § 5.

## 2 Simulation details

We numerically solve the three-dimensional incompressible Navier–Stokes equations within the Boussinesq approximation, which in non-dimensional form read 2.1
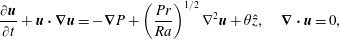

2.2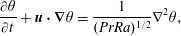 with 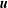 the velocity non-dimensionalized by the free-fall velocity 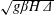, 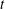 the time non-dimensionalized by 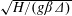, 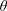 the temperature non-dimensionalized by the temperature difference between the plates 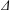 and 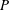 the pressure non-dimensionalized by 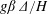. All our length scales are non-dimensionalized by 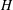, implying that we set the plate distance to unity in this work.

To solve (2.1) and (2.2) we employ the second-order finite difference code AFiD (van der Poel *et al.* 2015), which has been validated many times against other numerical and experimental results (Verzicco & Orlandi 1996; Verzicco & Camussi 1997, 2003; Stevens *et al.* 2010, 2011; Ostilla-Mónico *et al.* 2014; Kooij *et al.* 2018). The code uses periodic boundary conditions with uniform mesh spacing in the horizontal directions and supports a non-uniform grid distribution in the wall-normal direction. For this study, we used the GPU version of the code (Zhu *et al.* 2018*b*) to allow efficient execution of many large-scale simulations. The Couette flow forcing is realized by moving both walls in opposite directions with a velocity of 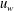, and the results for the classic Couette flow case match excellently with the results by Pirozzoli *et al.* (2014). For example, figure 2(*b*) shows that, for Couette flow, 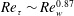, which agrees very well with the Couette data of Pirozzoli *et al.* (2014) and Avsarkisov *et al.* (2014).

**Figure 3.**
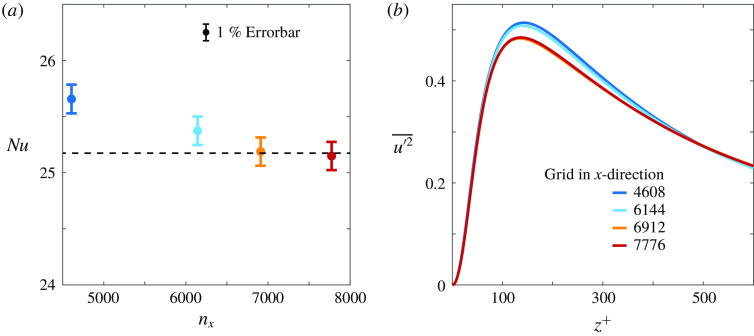
Values of (*a*) 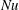 and (*b*) the streamwise velocity fluctuations for simulations at 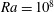 and 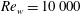 performed using different grid resolutions. The simulations are performed in a box of 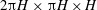 in the streamwise, spanwise and vertical directions, respectively. The displayed resolutions indicate the extrapolated streamwise resolutions that correspond to the full 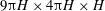 box, see table 1 for details. Note that the simulation results are converged for the grid resolution used in this study.

All simulations in this study were performed in a large 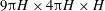 box, in the streamwise, spanwise and wall-normal directions (Tsukahara *et al.* 2006; Pirozzoli *et al.* 2014), which is required to capture the large-scale structures formed in Couette flow (Avsarkisov *et al.* 2014; Pirozzoli *et al.* 2014; Lee & Moser 2018). We adopted the grid distribution used by Pirozzoli *et al.* (2014, 2017), which is based on the resolution requirements for pure buoyant flow (Shishkina *et al.* 2010) and pure channel flow (Bernardini, Pirozzoli & Orlandi 2014), which is very similar to our flow configuration. The average horizontal grid spacing in the mixed convection simulations is 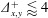. In the wall-normal direction the boundary layers are, on average, resolved up to 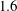 Kolmogorov lengths. For the case at 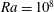 and 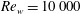, i.e. the most challenging simulation of this study, we used a horizontal grid spacing of less than three wall units in both horizontal directions. There are 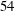 grid points in each boundary layer to ensure that the boundary layers are resolved up to 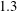 Kolmogorov lengths. We present a grid refinement check, which was performed in a smaller 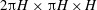 domain to keep the test manageable, for this case in table 1. Figure 3 confirms that the simulations are fully resolved for the chosen resolution. As further validation, we show in § 3.2 that our data excellently agree with the Couette data from Pirozzoli *et al.* (2014). We make sure that all simulations have reached the statistically stationary state before collecting data. Table 2 shows the simulation parameters for the main cases presented in this study.

**Table 1.** Grid convergence study for 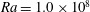, 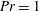 and 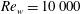 in a 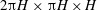 domain. 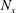, 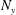, 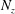 indicate the resolution in the streamwise 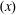, spanwise 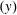 and wall-normal 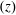 directions, respectively. The other columns show the friction Reynolds number 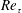, the Monin–Obukhov length 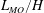, the Nusselt number 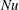 and the friction coefficient 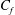. Additional information on the grid convergence study is provided in figure 3.

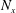	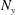	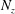	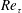	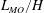	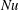	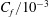
1024	512	384	708.0	0.138	25.66	10.03
1296	648	384	703.6	0.137	25.37	9.902
1536	768	384	700.6	0.137	25.19	9.818
1728	864	384	700.0	0.136	25.15	9.805

**Table 2.** Main simulations considered in this work.

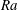	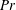	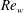	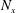	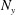	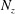	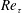	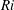	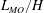	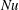	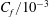
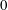	1	2000	1280	1024	256	131.9	0	0	0	8.695
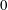	1	3000	1280	1024	256	185.8	0	0	0	7.673
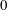	1	4000	1280	1024	256	238.1	0	0	0	7.089
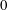	1	6000	1280	1024	256	340.6	0	0	0	6.446
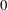	1	8000	2048	1280	256	439.7	0	0	0	6.041
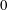	1	10 000	3072	1536	256	540.9	0	0	0	5.852
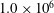	1	0	1280	1024	256	—	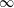	0	8.343	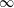
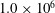	1	2000	1280	1024	256	161.7	0.250	0.645	6.557	13.07
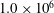	1	3000	1280	1024	256	203.0	0.111	1.218	6.869	9.158
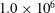	1	4000	1280	1024	256	251.7	0.063	2.022	7.891	7.922
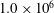	1	6000	2048	1280	256	355.2	0.028	4.259	10.52	7.008
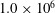	1	8000	3072	1536	256	452.2	0.016	7.212	12.82	6.390
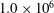	1	10 000	4608	2304	320	554.3	0.010	11.00	15.49	6.145
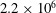	1	0	1280	1024	256	—	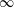	0	10.40	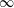
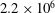	1	2000	1280	1024	256	176.3	0.550	0.316	7.866	15.53
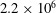	1	3000	1280	1024	256	215.3	0.244	0.583	7.788	10.30
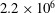	1	4000	1280	1024	256	261.9	0.138	0.953	8.568	8.576
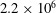	1	6000	2048	1280	256	363.0	0.061	1.983	10.96	7.319
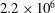	1	8000	4096	2048	256	462.8	0.034	3.351	13.44	6.692
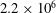	1	10 000	4608	2304	320	566.4	0.022	5.123	16.12	6.416
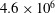	1	0	1280	1024	256	—	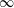	0	12.83	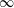
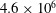	1	2000	1280	1024	256	190.2	1.150	0.156	9.353	18.09
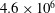	1	3000	1280	1024	256	237.3	0.511	0.306	9.502	12.51
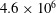	1	4000	1280	1024	256	276.1	0.288	0.475	9.626	9.526
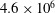	1	6000	2048	1280	256	377.2	0.128	0.982	11.88	7.903
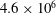	1	8000	4096	2048	256	474.0	0.072	1.645	14.08	7.021
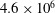	1	10 000	4608	2304	320	578.7	0.046	2.513	16.76	6.697
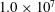	1	0	1280	1024	256	—	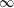	0	16.18	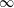
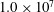	1	2000	1280	1024	256	213.2	2.500	0.078	12.41	22.73
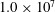	1	3000	1280	1024	256	265.8	1.111	0.156	12.02	15.69
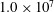	1	4000	1280	1024	256	306.0	0.625	0.243	11.78	11.70
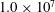	1	6000	2048	1280	256	391.2	0.278	0.466	12.85	8.502
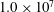	1	8000	4096	2048	256	493.4	0.156	0.785	15.30	7.607
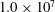	1	10 000	6144	3072	320	595.0	0.100	1.180	17.85	7.080
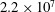	1	0	3072	1536	256	—	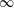	0	19.86	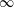
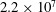	1	2000	3072	1536	256	236.4	5.500	0.036	16.62	27.94
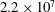	1	3000	3072	1536	256	296.5	2.445	0.075	15.77	19.53
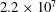	1	4000	3072	1536	256	341.4	1.375	0.119	15.24	14.57
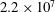	1	6000	3072	1536	256	429.1	0.611	0.233	15.43	10.23
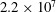	1	8000	4096	2048	256	514.0	0.344	0.374	16.52	8.255
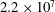	1	10 000	6144	3072	320	607.6	0.220	0.552	18.01	7.383
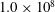	1	0	4096	2048	256	—	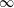	0	31.14	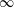
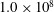	1	2000	4096	2048	256	283.9	25.00	0.007	29.34	40.31
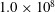	1	3000	4096	2048	256	360.1	11.11	0.017	27.85	28.82
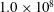	1	4000	4096	2048	256	425.7	6.250	0.029	26.83	22.66
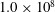	1	6000	4608	2304	320	534.7	2.778	0.059	25.81	15.88
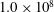	1	8000	6144	3072	320	628.5	1.563	0.097	25.72	12.35
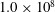	1	10 000	6912	3456	384	713.3	1.000	0.139	26.03	10.18

## 3 Global flow characteristics

### 3.1 Effective scaling of the Nusselt number

**Figure 4.**
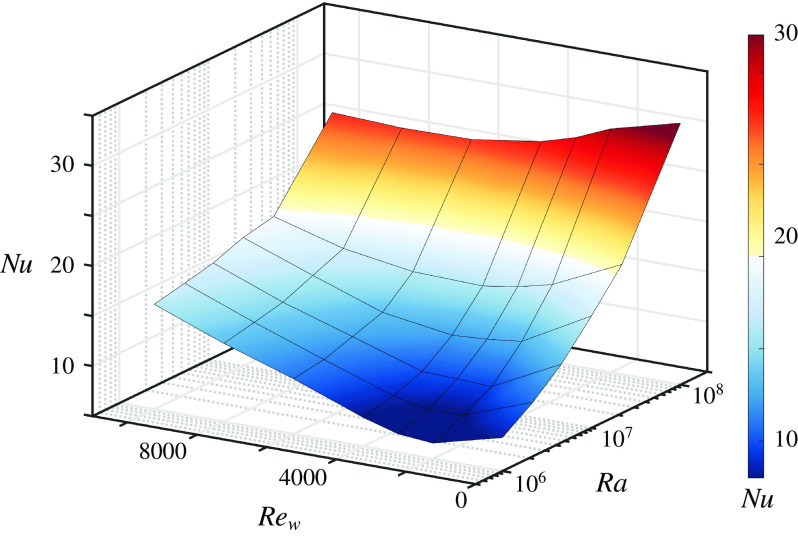
Value of 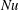 as a function of 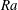 and 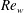 in Couette–RB flow.

**Figure 5.**
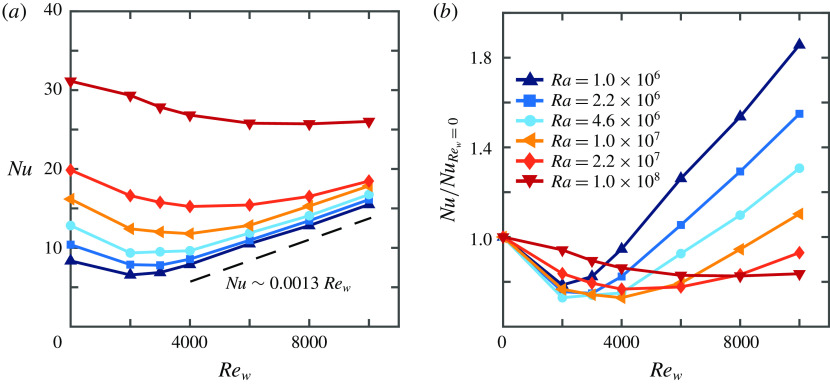
Values of (*a*) 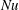 and (*b*) 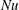 normalized by the RB value 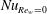 as a function of 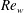.

Figure 4 shows that the heat transfer increases with increasing 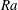 and 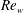 and that for a given 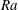 number a minimum heat transfer is obtained at some intermediate 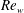. Scagliarini *et al.* (2014) showed that the minimum is caused by the thermal plumes being swept away by the shear. Figure 5(*a*) shows cross-sections for constant 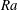 which reveal that the location of the minimum heat transfer at constant 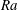 shifts towards higher 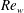 with increasing 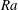. For high enough 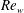, the behaviour of 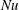 converges towards 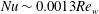. Figure 5(*b*), where 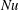 is normalized by the RB value for the respective 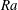, shows that the drop in 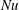 becomes less pronounced and is observed at higher 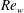 when 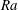 is increased. This is a good indication that the thermal plumes become stronger and therefore harder to disturb by the applied shear. For 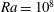 the decrease in 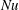 at 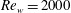 is only 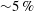 while the data for other 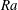 show percentages up to the high twenties. A more detailed analysis would need more data points for low 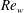, which are difficult to obtain due to the computational time that is required for each simulation.

**Figure 6.**
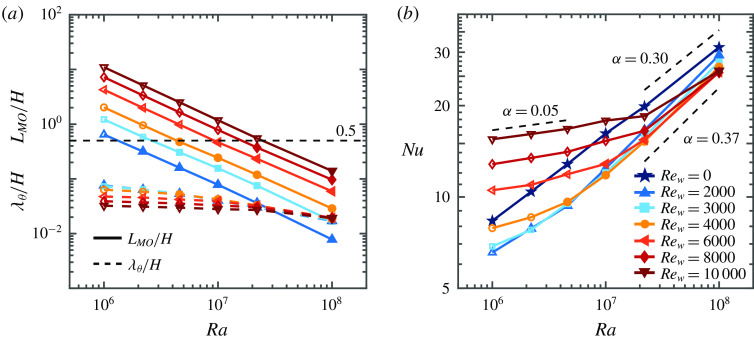
(*a*) The Monin–Obukhov length as a function of 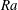 for different 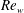. The Monin–Obukhov length (solid lines) is compared to the thermal boundary layer thickness (dashed lines) and to 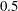 to define the flow regime (buoyancy dominated, transitional, shear dominated) of each simulation, see details in the text. Open symbols indicate 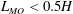. (*b*) 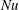 as a function of 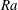. The numbers indicate the scaling exponent 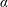 in 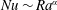. The 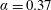 effective scaling line is plotted for visual reference only.

The results indicate that the heat transfer is influenced by the ratio of the buoyancy and shear forces. Therefore, the bulk Richardson number 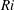 or the above-defined Monin–Obukhov length, which take the ratio of these forces into account, are natural control and response parameters to identify the different flow regimes. Although the Monin–Obukhov theory itself is only valid for shear dominated flow, which does not necessarily exist in all our simulations, we use the Monin–Obukhov length as an objective criterion to distinguish between buoyancy and shear-driven flow. This choice builds on a long and rich tradition of using the Monin–Obukhov length to characterize mixed convective flows, namely in the seminal works by Obukhov (1946) and Monin & Obukhov (1954). Its use has significantly advanced the understanding of mixed convective flows. The Monin–Obukhov length is relevant as it characterizes the effects of both friction and buoyancy, the main physical effects in this system, by a single length scale. Also, in this case, we show that the Monin–Obukhov length is a relevant parameter that gives insight into the behaviour of the flow. From the data in table 2, we find 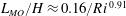 (see appendix C). In figure 6(*a*) the Monin–Obukhov length is compared to the thermal boundary layer thickness 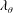, which is determined from 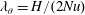. Since 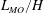 is the fraction of the domain in which the shear forcing is dominant 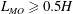 indicates when the flow is completely shear dominated. This allows us to define three different flow regimes, namely a buoyancy dominated regime (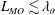), a transitional regime (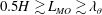) and a shear dominated regime (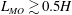). At the moment we cannot more accurately determine the Monin–Obukhov length at which the transition takes place, but the presented ranges provide a good indication of the required thermal and shear forcing to achieve the different regimes. A similar behaviour has also been observed in convective boundary layers, where Salesky, Chamecki & Bou-Zeid (2017) find a cell dominated regime for 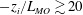, where 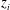 is the convective boundary layer thickness, a cell and roll dominated regime as transitional state, and a roll dominated regime for 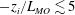.

Figure 6(*b*) shows that the heat transfer in the buoyancy dominated regime scales as 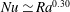, which is in agreement with results for classical RB convection (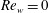, Ahlers *et al.* (2009)). For the shear dominated regime we find that the effective scaling exponent 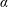 in 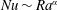 is 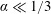 and in the transitional regime we find 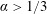. An effective scaling exponent larger than 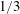 is one of the characteristics of the ultimate regime. It should occur when the boundary layers have transitioned to the turbulent state, which is indicated by their logarithmic profiles. Our analysis in § 4 shows that this is not yet the case in this transitional regime. Instead, for intermediate shear, the heat transfer is decreased with respect to the RB case. The locally larger effective scaling exponent simply is a consequence of the fact that with increasing 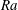 the heat transfer, which was decreased at intermediate shear, must again converge to the RB case.

### 3.2 Skin friction

**Figure 7.**
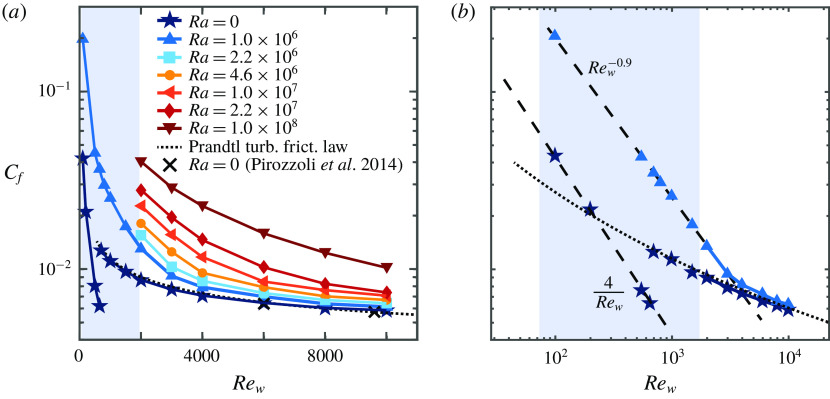
(*a*) Skin friction coefficient 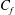 as a function of 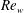. (*b*) Zoom in of the grey area shown in panel (*a*), now on a logarithmic scale, showing the data for pure Couette flow (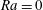, stars) and 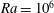. Note that 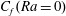 follows the expected laminar result (- - -) until 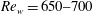 and then jumps to the turbulent curve (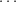). For Couette–RB, i.e. the up-pointing triangle, no jump is observed.

In figure 7 we compare the measured skin friction coefficient for different 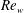 and 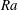 with Prandtl’s turbulent friction law (Schlichting & Gersten 2000) 3.1
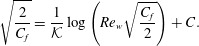



**Figure 8.**
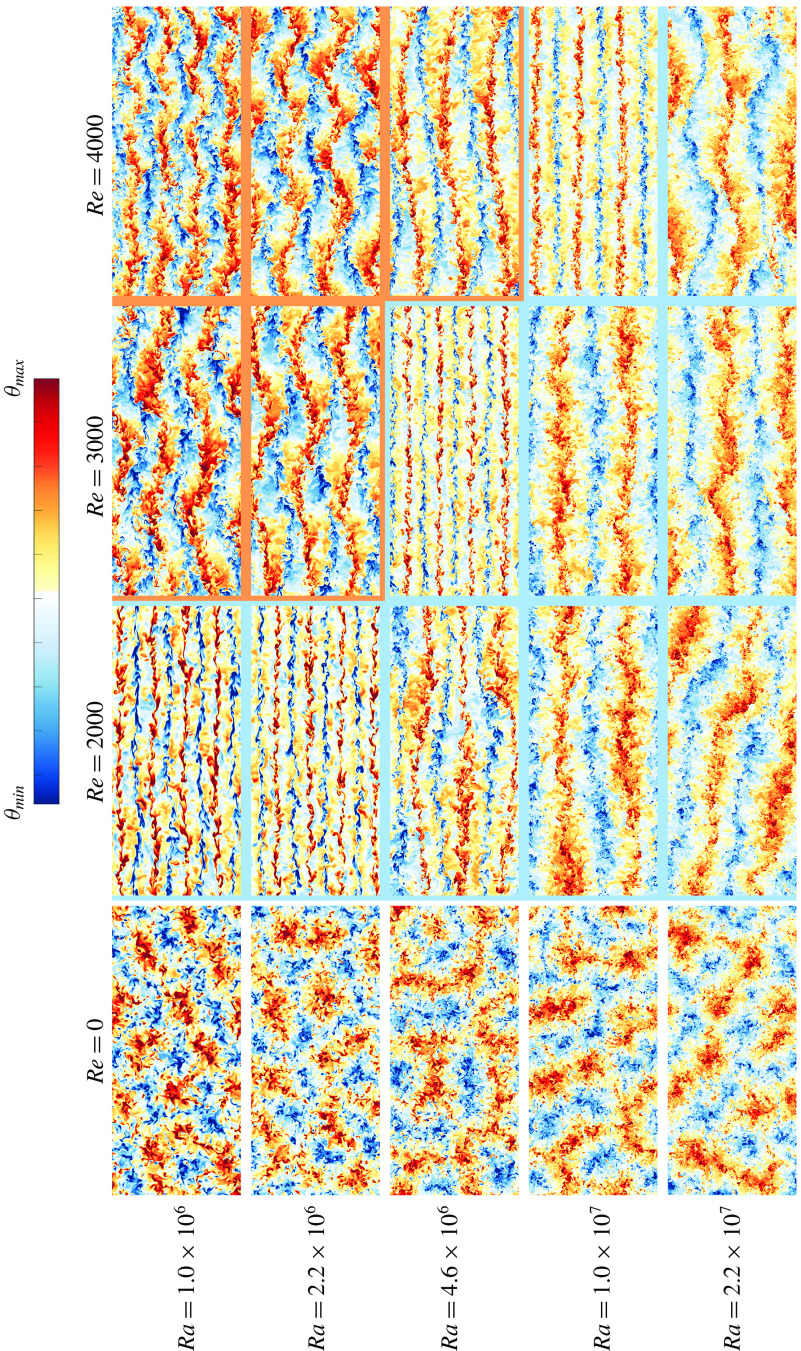
Instantaneous snapshots of temperature fields at mid-height for a subdomain of the parameter space, see figure 2(*a*) and table 2, focusing on 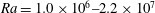 and 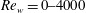. The panels have coloured borders depending on the flow regime they display: buoyancy dominated (white), transitional (blue) and shear dominated (orange) regime. For a more detailed quantification of the different flow fields in the presented snapshots, we refer to the values for the Monin–Obukhov length in table 2. An overview of temperature fields over the whole domain can be found in appendix A. The colour range for the snapshots in this figure and in figures 9–11 and 14 is adjusted such that the most important thermal structures are clearly visible.

**Figure 9.**
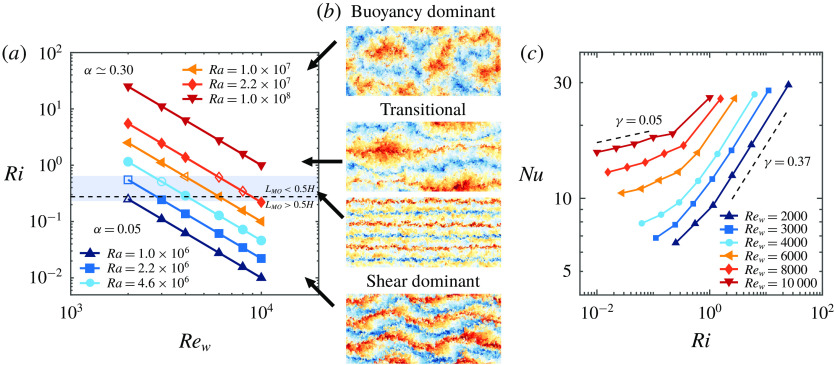
(*a*) 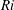 versus 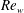 for different 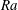. Open symbols indicate the presence of thin straight elongated streaks (see third snapshot from top). The dashed line indicates 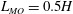. In (*b*) instantaneous snapshots of temperature fields at mid-height. (*c*) 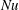 as a function of 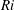 for different 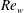. An indication of the effective scaling exponent 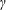 in 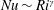 in the different regimes is also given. For a more detailed quantification of the different flow regimes in the presented snapshots, we refer to the Monin–Obukhov lengths documented in table 2.

Following Pirozzoli *et al.* (2014) we use a von Kármán constant 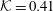 and 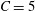. The figure shows that the skin friction increases with 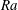 and decreases with 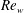. At fixed 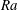 the relative strength of the thermal forcing decreases for high 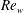, and therefore the obtained friction coefficient converges to the Prandtl law. This agrees very well with the findings of Scagliarini *et al.* (2015) and Pirozzoli *et al.* (2017) for Poiseuille–RB flow. In figure 7(*b*) we focus on the data for small 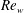. The skin friction in pure Couette flow follows the expected laminar result 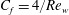 (Pope 2000) until a transition to the turbulent state occurs around 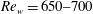. Cerbus *et al.* (2018) discuss that in pipe flow this jump is caused by the formation of puffs and slugs. Brethouwer, Duguet & Schlatter (2012) attribute this discontinuous jump in 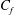 to the lack of restoring forces in plane Couette flow (similar to pipe, channel, and boundary layer flows). For the Couette–RB case we do not observe such a discontinuous jump. Instead, this sheared RB case is another example, next to the application of Coriolis, buoyancy and Lorentz forces discussed by Brethouwer *et al.* (2012), which shows that restoring forces can prevent a discontinuous jump in 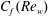. Chantry *et al.* (2017), on the other hand, claim that all transitions to turbulence should be continuous if the used box size is large enough. From this figure we can also judge whether the boundary layer is turbulent or not. When the slope of 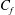 approaches the one of pure Couette flow the boundary layers are turbulent. We consider the boundary layer as non-turbulent when this slope starts to strongly deviate from the Prandtl law.

**Figure 10.**
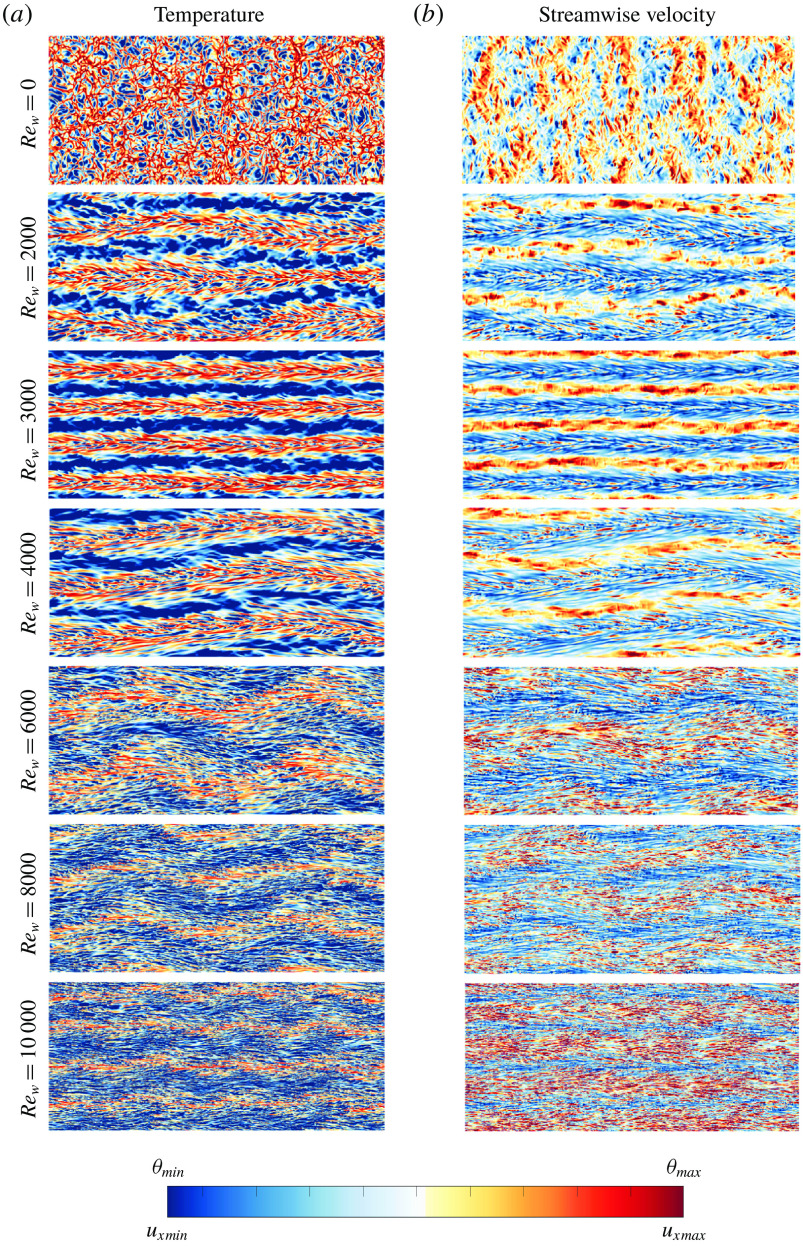
Instantaneous near-wall snapshots at 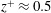 of the temperature (*a*) and streamwise velocity (*b*) for 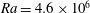. 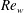 increases from top to bottom. For a more detailed quantification of the different flow fields in the presented snapshots, we refer to the values for the Monin–Obukhov length in table 2. The colour range for the snapshots in this figure is adjusted such that the most important thermal structures are clearly visible.

## 4 Local flow characteristics

### 4.1 Organization of turbulent structures

To further investigate the dynamics of the different regimes we show visualizations of the temperature field for all simulations in figure 8 and appendix A. We decided to show the flow at mid-height because there the flow is least affected by the walls. In the buoyancy dominated regime the primary flow structure resembles the large-scale flow found in RB convection (Stevens *et al.* 2018). In the transitional regime (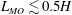), the thermal forcing dominates part of the bulk where large elongated thermal plumes transform into thin straight elongated streaks when 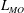 approaches 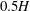. In figure 8 and in appendix A this manifests itself as a very visual line diagonally through the diagram, splitting the more thermal and the more shear dominated cases. In the shear dominated regime (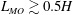) we find large-scale meandering structures, similar to the ones found in pressure-driven channel flow with unstable stratification (Pirozzoli *et al.* 2017). This significant change in flow structure can be linked to the minimum in 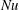 in figure 5. The reason for the minimum is that at intermediate shear the thermal convection rolls are broken up, while the shear is not yet strong enough to increase the heat transfer directly. This observation is in agreement with earlier works described above (Domaradzki & Metcalfe 1988; Scagliarini *et al.* 2014, 2015; Pirozzoli *et al.* 2017).

In figure 9 we present a clear overview of the behaviour of the flow structures versus the flow control parameters combined in the bulk Richardson number. In panel (*a*), we compare the different values of 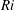 with the visually observed flow structures. We find a range of 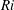 in which the flow undergoes a change from the transitional to the shear dominated regime. This happens in a range of 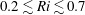. In panel (*c*), we can also detect this trend, where the effective scaling exponent 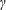 in 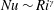 changes from 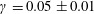 to 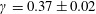. We note that more data points would be necessary to define the transition point more accurately.

Figure 9 combines these findings with the above observation that in the shear dominated regime the effective scaling exponent 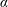 in 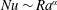 is much smaller than 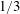, in the transitional regime 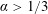, and in the buoyancy dominated regime 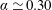. When we compare the regime transitions with the results in figure 5 it becomes clear that the lowest heat transfer for a given 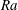 occurs at the end of the transitional regime before the emergence of the thin straight elongated streaks. Due to the large computational time that is required for each simulation the number of considered cases is limited, which makes it difficult to pinpoint exactly when the heat transfer is minimal and what the flow structure looks like in that case. However, we note that the onset of the shear dominant regime corresponds to the point where the heat transfer starts to increase as the additional shear can then more effectively enhance the overall heat transport.

To get more insight into the boundary layer dynamics in the different regimes, we show the temperature and streamwise velocity at the boundary layer height for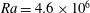 in figure 10. At this Rayleigh number the flow is in the transitional regime for 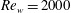 and 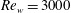, and in the shear dominant regime for 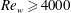. For all cases we observe a clear imprint of the large-scale structures observed at mid-height, see figure 8 and appendix A. This indicates that the large-scale dynamics has a pronounced influence on the flow structures in the boundary layers (Stevens *et al.* 2018). The figure also reveals that in the transitional and shear dominated regimes the lowest temperatures at boundary layer height are observed in the high-speed streak regions, which indicates that the regions with the highest shear contribute most to the overall heat flux.

### 4.2 Flow statistics

**Figure 11.**
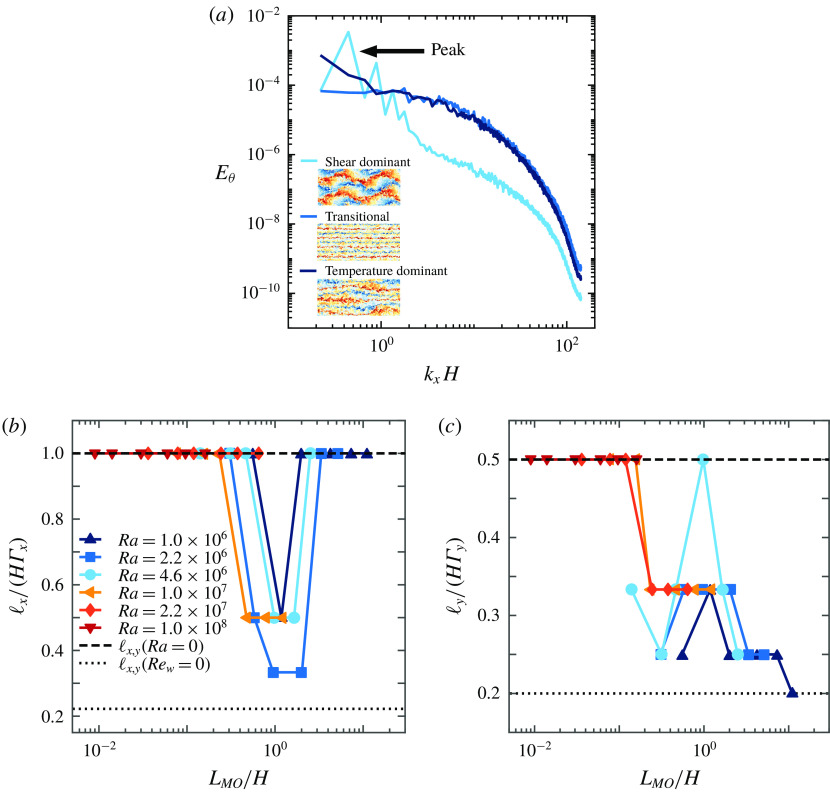
(*a*) Overview of streamwise temperature variance spectra at mid-height at 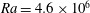 for the different flow regimes. Panels (*b*,*c*) show the evolution of peaks in streamwise and spanwise temperature variance spectra, respectively, versus the Monin–Obukhov length. The spectra were evaluated on three-dimensional snapshots and are subsequently time averaged.

We now present the streamwise temperature variance spectra 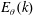 in figure 11 to analyse the size of the large-scale structures as function of the Monin–Obukhov length. The position of the peak in the temperature spectrum indicates the wavelength of the most prominent thermal structure (Stevens *et al.* 2018). In panels (*b*,*c*) we plot the evolution of these wavelengths versus the absolute size of the flow domain. Therefore we define 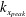 and 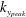 as the wavenumbers of the peak in the respective energy spectrum and 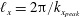 and 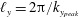 as the respective wavelengths. If the spectrum does not show a clear peak but keeps growing for small 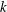, the structure size is limited by the box size, which is 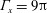 in streamwise (figure 11 *b*) and 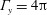 in spanwise direction (figure 11 *c*).

For 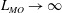, 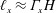, which is expected since for pure Couette flow, structures much larger than 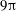 are expected (Lee & Moser 2018). 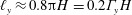 for the highest shear case, but here more data points are needed for a clearer determination of its behaviour. In the other limit of 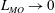, i.e. in the transitional regime as the RB case (buoyancy dominated regime) is not shown due to the logarithmic axis, the large-scale structures are elongated over the whole streamwise length, which is consistent with figures 8 and 14. For pure RB convection, where 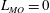, 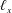 decreases to 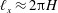, which is in agreement with Stevens *et al.* (2018). In the spanwise direction, the flow converges already much earlier to the RB case where 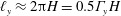. In the shear dominated regime, where the flow meanders, the structure size in streamwise direction drops to about half the box length. In the spanwise direction, this flow regime is present as a local peak in panel (*c*). Due to the minimal number of data points, it is not possible to fully assess the behaviour of 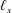 and 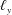 versus 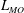 for all 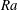 and 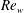. Nevertheless, the minimum in 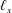 and peak in 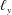 in the shear dominated regime are very distinct.

**Figure 12.**
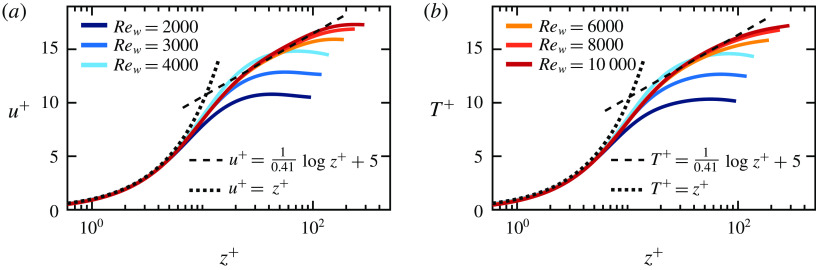
(*a*) Mean streamwise velocity and (*b*) temperature profiles, where 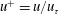 and, following Pirozzoli *et al.* (2017), 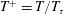 with the friction temperature 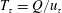 for 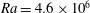.

To further quantify the cases shown in figure 10, in figure 12 we show the streamwise velocity and temperature profiles for fixed 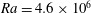 and increasing 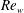 from 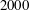 to 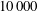. As can be seen, for the wall Reynolds number in the transitional range up to 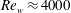 (see corresponding curve in figure 9 *a*) neither the temperature nor the streamwise velocity profiles are logarithmic. This indicates that the boundary layers are not yet turbulent in this state. Hence, the higher 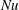 scaling in this transitional regime is not caused by a triggered transition to the ultimate regime. Note that spatio-temporally chaotic flow with thermal plume detachment from the walls must not be confused with turbulent flow. The temporal fluctuations in this regime can in fact be considerable, but given the small shear Reynolds numbers, the flow is still not turbulent. Here we use the definition of turbulence as absence of large-scale coherence in space and time.

In contrast, in the shear dominated regime (beyond 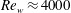 for this 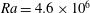) the streamwise velocity and temperature profiles do start to converge to a logarithmic profile with further increasing 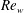, reflecting the onset of the ultimate regime. This observation is in agreement with previous findings for Couette–RB (Liu 2003; Choi, Chung & Kim 2004; Debusschere & Rutland 2004; Le & Papavassiliou 2006) and Poiseuille–RB (Scagliarini *et al.* 2015; Pirozzoli *et al.* 2017).

**Figure 13.**
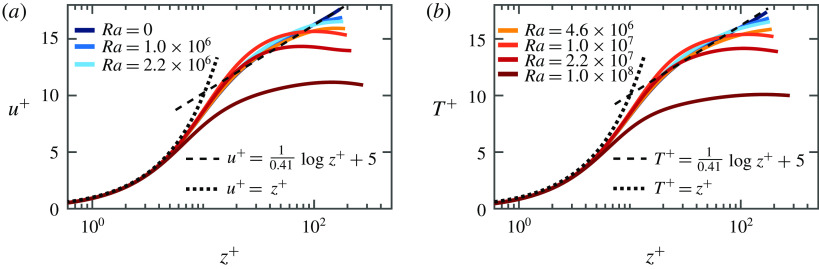
(*a*) Mean streamwise velocity and (*b*) temperature profiles, where 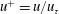 and 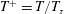 with 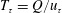 for 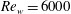. 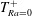 was determined through a passive-scalar temperature field.

In figure 13 we show the same statistical quantities as in figure 12, but now for fixed 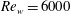. For 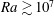 the flow is in the transitional regime and for 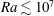 the flow undergoes a transition into the shear dominated regime. Just as in figure 12 we observe that the temperature and streamwise velocity profiles are not logarithmic in the transitional regime. As the Richardson number decreases with decreasing 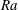, we see that the profiles converge towards a logarithmic behaviour. From a comparison with table 2 we find that 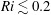 seems to be required to achieve logarithmic temperature and velocity profiles. A comparison with the results shown in figure 9 confirms that 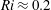 is indeed the threshold where the flow undergoes its transition to the shear dominated regime. This is also consistent with the work of Pirozzoli *et al.* (2017), who report a regime with the increased importance of friction at 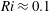. For the parameter regime under investigation, the effective scaling exponent 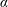 in the shear dominated regime is well below 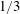. In both figures we can detect a non-monotonic behaviour of both 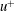 and 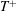 for low 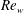 and high 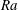. The non-monotonic temperature profile indicates the formation of different flow layers, i.e. heat that is carried by hot plumes originating from the bottom plate gets entrapped somewhere in the middle of the domain. Similarly, some of the cold plumes originating from the top plate also get trapped. Several further statistical quantities are presented in appendix B.

## 5 Concluding remarks

We performed direct numerical simulations of turbulent thermal convection with Couette type flow shearing. We presented cases in a range 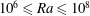 and 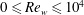, achieving up to 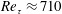. For fixed Rayleigh number we obtain a non-monotonic progression of 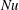 similarly to what was previously observed in unstable stratification with a pressure gradient (Scagliarini *et al.* 2014). The addition of imposed shear to thermal convection first leads to a reduction of the heat transport by disrupting the turbulent system before the shear becomes strong enough to create meandering streaks that efficiently transport the heat away from the wall. As the impact of the thermal plumes on the flow decreases with increasing shear, the skin friction coefficient at constant 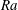 drops with increasing 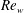.

Using the Monin–Obukhov length 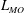 and the thermal boundary layer thickness 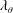, we identify three flow regimes. In the buoyancy dominated regime (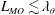) large thermal plumes dominate the flow. With decreasing Richardson number we first find a transitional regime (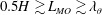), before the shear dominated flow regime with large-scale meandering streaks is obtained. For given 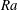 the minimum heat transport is found before the onset of this shear dominated regime when thin straight elongated streaks dominate the flow. We find that in the transitional regime the effective scaling exponent 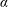 in 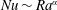 is larger than 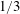. An analysis of the flow characteristics shows that the temperature and streamwise velocity profiles are not logarithmic in this transitional regime, which one would expect when this high scaling exponent would indicate the onset of the ultimate regime. We want to investigate in future studies whether it is possible to further increase the thermal and sheared forcing far enough to trigger the occurrence of a logarithmic velocity profile in the boundary layer and thus the ultimate convection in Couette–RB, but considerably more CPU time is required for that.

**Figure 14.**
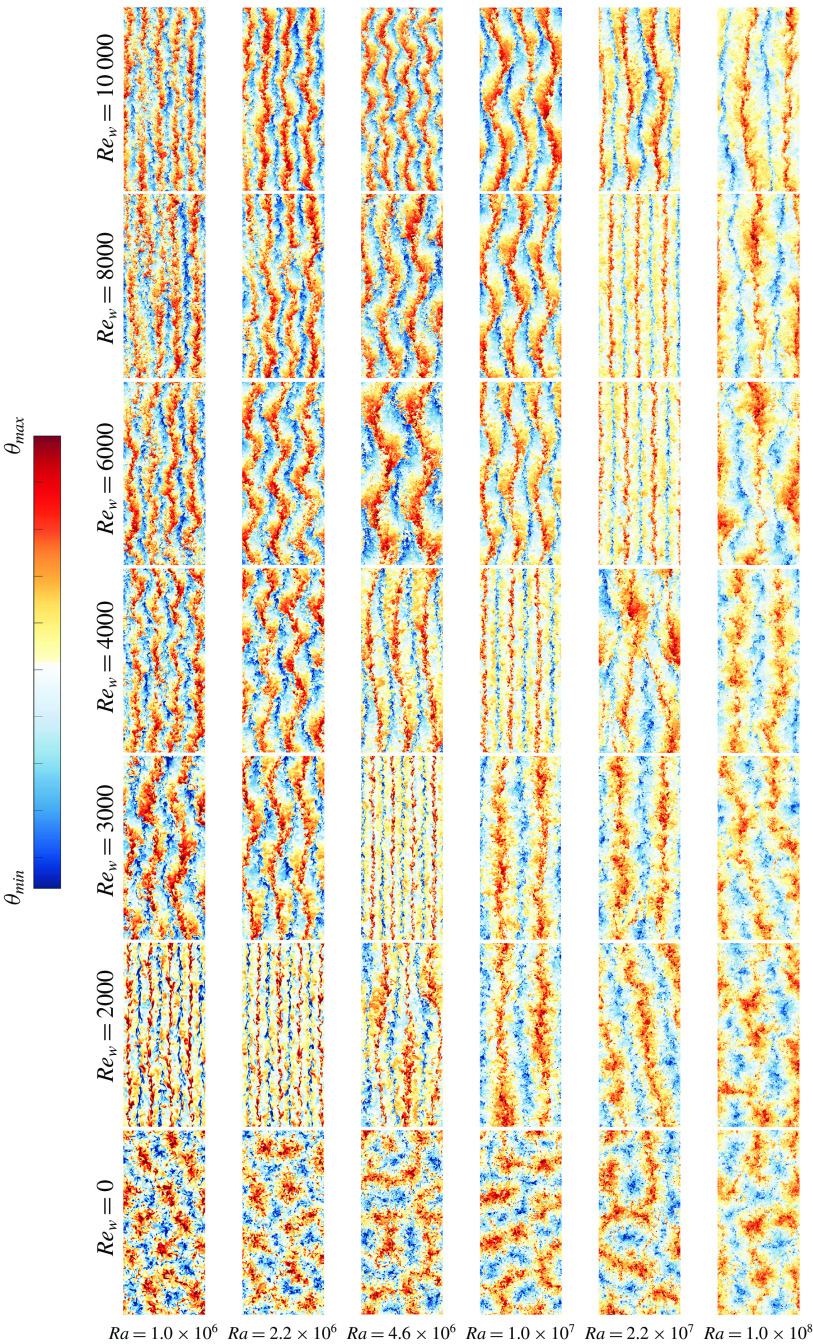
Instantaneous snapshots of all simulated temperature fields at mid-height, see the caption of figure 8 for further details.

## Figures and Tables

**Figure 15.**
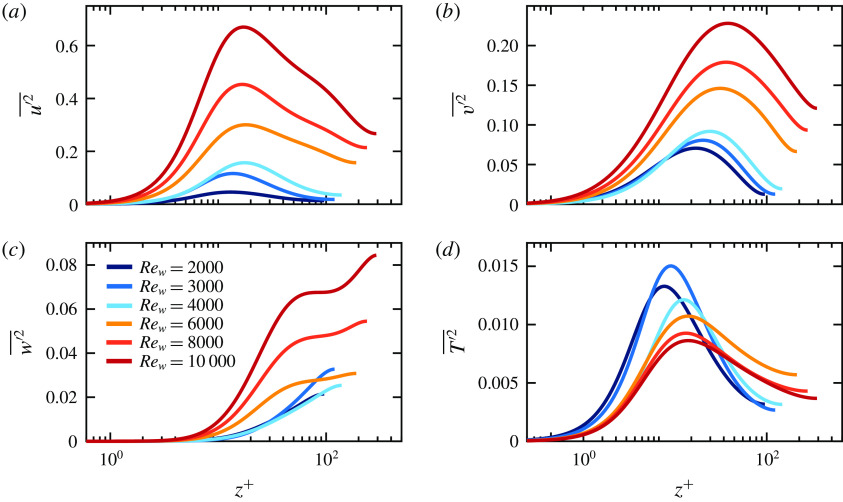
Fluctuations of (*a*) streamwise, (*b*) spanwise and (*c*) wall-normal velocities, and (*d*) temperature for 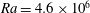.

**Figure 16.**
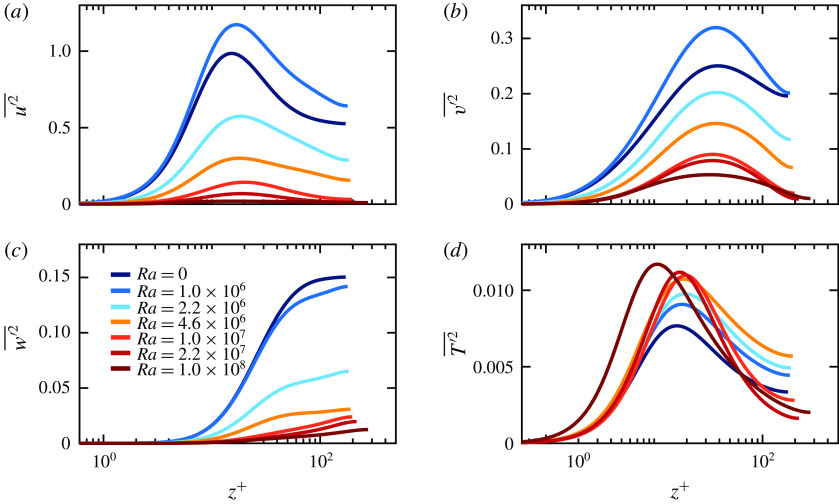
Fluctuations of (*a*) streamwise, (*b*) spanwise and (*c*) wall-normal velocities, and (*d*) temperature for 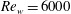. 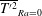 was determined through a passive-scalar temperature field.

**Figure 17.**
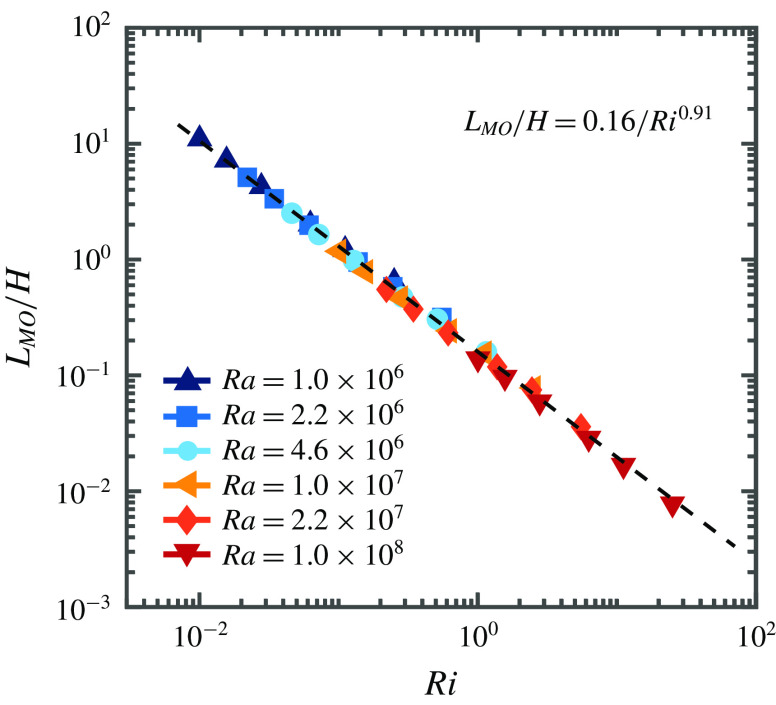
Value of 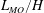 versus 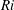 and the corresponding fit 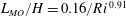 to the data.

**Figure 18.**
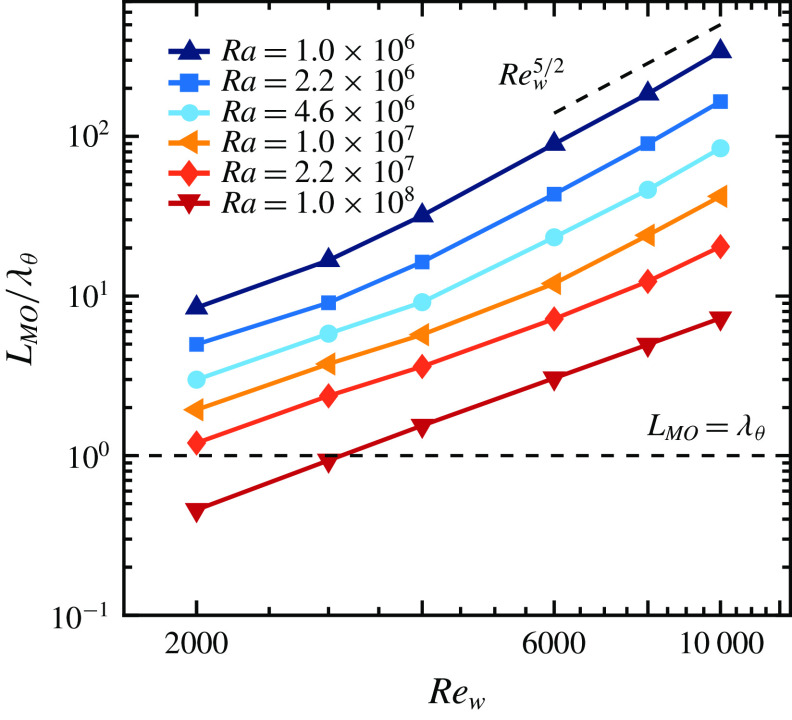
Value of 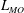 normalized by the thermal boundary layer thickness 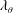 versus 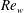. For 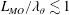 the flow is in the buoyancy dominated regime.

## Appendix A. Flow field overview

As an addition to figure 8 we present in figure 14 the full overview of temperature snapshots at mid-height, ranging from  and . All three regimes, i.e. the buoyancy dominated regime, the transitional regime and the shear dominated regime, can be observed here.

## Appendix B. Further flow statistics

In addition to the data presented in figures 12 and 13 we present further flow statistics here. Figure 15 shows the velocity and temperature fluctuations as function of height for  for various wall shear Reynolds numbers. It can be observed that the velocity fluctuations increase with . The temperature fluctuations show a non-monotonic behaviour. The peak temperature fluctuations first increase with increasing  before the peak of the temperature fluctuations decreases with increasing wall shear. Figure 16 shows the velocity and temperature fluctuations for  and increasing .  and  first increase and then monotonically decrease with increasing  when thermal forcing is added to Couette flow. Both the wall-normal velocity and the temperature fluctuations decrease completely monotonically for increasing thermal forcing.

## Appendix C. Scaling of the Monin–Obukhov length with Richardson number

In figure 17 we present the Monin–Obukhov length  versus the Richardson number  for all simulations. We find that the Monin–Obukhov length scales as , similar to  as found by Pirozzoli *et al.* (2017) for channel flow with unstable stratification.

## Appendix D. Comparison of and

In figure 18 we show the ratio between the Monin–Obukhov length  and the thermal boundary layer thickness , which is determined from . For  the flow is in the buoyancy dominated regime. For higher , the flow first reaches the transitional regime before the shear dominated regime is reached, where .
